# Sustainable drug release from polycaprolactone coated chitin-lignin gel fibrous scaffolds

**DOI:** 10.1038/s41598-020-76971-w

**Published:** 2020-11-24

**Authors:** Turdimuhammad Abdullah, Kalamegam Gauthaman, Azadeh Mostafavi, Ahmed Alshahrie, Numan Salah, Pierfrancesco Morganti, Angelo Chianese, Ali Tamayol, Adnan Memic

**Affiliations:** 1 Center of Nanotechnology, King Abdulaziz University, Jeddah, Saudi Arabia; 2 Center of Excellence in Genomic Medicine Research, King Abdulaziz University, Jeddah, Saudi Arabia; 3 Faculty of Medicine, AIMST University, Semeling, Bedong, Kedah Malaysia; 4 Department of Mechanical and Materials Engineering, University of Nebraska, Lincoln, NE USA; 5 Academy of History of Healthcare Art, Rome, Italy; 6 ISCD Nanoscience Center, Rome, Italy; 7 LABOR Srl, Rome, Italy; 8 Department of Biomedical Engineering, University of Connecticut Health Center, Farmington, CT 06030 USA

**Keywords:** Biochemistry, Biotechnology, Microbiology, Medical research, Molecular medicine, Materials science, Nanoscience and technology

## Abstract

Non-healing wounds have placed an enormous stress on both patients and healthcare systems worldwide. Severe complications induced by these wounds can lead to limb amputation or even death and urgently require more effective treatments. Electrospun scaffolds have great potential for improving wound healing treatments by providing controlled drug delivery. Previously, we developed fibrous scaffolds from complex carbohydrate polymers [i.e. chitin-lignin (CL) gels]. However, their application was limited by solubility and undesirable burst drug release. Here, a coaxial electrospinning is applied to encapsulate the CL gels with polycaprolactone (PCL). Presence of a PCL shell layer thus provides longer shelf-life for the CL gels in a wet environment and sustainable drug release. Antibiotics loaded into core–shell fibrous platform effectively inhibit both gram-positive and -negative bacteria without inducting observable cytotoxicity. Therefore, PCL coated CL fibrous gel platforms appear to be good candidates for controlled drug release based wound dressing applications.

## Introduction

Diabetic ulcers and other chronic wounds are a major healthcare burden and a source of staggering costs in both developing and developed countries^1^. Chronic wound often exhibit a hostile microenvironment characterized with elevated local pH levels, the excessive presence of degradative enzymes, and limited nutrient supply^1^. Such environments prevent tissue regeneration while supporting the growth of pathogens leading to serious infection^2,3^. Treatment of chronic wounds requires effective drug delivery systems that can stimulate physiological processes at the right time. Such drug delivery systems should take into consideration the various, concurrent physiological processes that lead to tissue regeneration^2,3^. Developing biomaterials that can both serve as wound dressings and control the release of drugs could improve treatment outcomes while simultaneously minimizing negative side effects^1,3^. For example, one of the most critical challenges in wound healing management is controlling bacterial infections^4,5^. Traditionally oral and intravenous administrations of antibiotics have been commonly used for infection treatment^2^. However, the localized delivery of antibiotics targets only the wound area representing a more promising approach to fighting infection, preventing drug resistance and lowering the risk of adverse side effects.

In recent years, electrospinning has gained much popularity for wound healing and other bioengineering applications^6,7^. It is a versatile technique to produce fibrous scaffolds with diameters from sub-nanometer to several micrometers, which can mimic properties of the native skin extracellular matrix (ECM)^8–10^. Morphology and size dimension of the electrospun fibers can be governed by numerous parameters including polymer concentration, solution viscosity, applied voltage, feed rate and humidity^11^. In addition, growth factors and drugs can be encapsulated due to high surface area and porosity of the electrospun meshes^12–14^. In electrospinning, the drugs can be uniformly dispersed into the nano/micro sized fibrous polymer mesh without aggregation and quantitative loss^11,15^. Moreover, adaptability of the electrospinning facilitates desirable drug loading/release by providing different drug loading approaches^13,16–18^.

Various types of gels have been electrospun for wound dressing applications including sol-gels and hydrogels that are all meant to adsorb and retain large amounts of water^8^. Hydrogels have emerged as promising wound dressing materials because they can fill irregular defects, provide a moist wound environment, serve as a barrier to microorganisms and deliver therapeutic agents to the injury site^19,20^. Additionally, they are typically formed from aqueous solutions, which prevents denaturation and aggregation of the loaded drugs upon exposure to organic solvents^19^. Particularly, natural polymer derived hydrogels have been highly pursued in wound healing applications due to their intrinsic biocompatibility, biodegradability, hemostatic property, antibacterial activity and stimulation of wound healing^8^.

Natural polymers such as chitin and lignin are widely available from food and agricultural by-products that can be made into gels^21,22^. Among them, chitin is a polysaccharide consisted of a long chain *N*-acetylglucosamine^23,24^. It is the second most available natural polymer in the world as the foremost component of crustacean shells and fungi cell wall^24^. Lignin, on the other hand, is a cross-linked phenolic biopolymer^24^. It is abundantly available as a by-product of pulp and the paper industry^24,25^. Annual global production of chitin is estimated to be about 100 billion tons, yet the commercialized amount of chitin is only 150,000 t^26^. Likewise, more than 300 billion tons of lignin are globally produced out of which only 2% are used to produce commercial products, and the rest are commonly used as fuel to obtain energy^27^. These polymers have numerous interesting characteristics that make them attractive for biomedical applications^28^. Chitin has moisturizing properties, super swelling capacity and anti-inflammatory activity; and lignin has antimicrobial activity as well as the ability to adsorb UV^29–31^. Their combination as a composite gel could entrap and release many bioactive compounds due to bivalency between positively charged chitin and negatively charged lignin^24,32^. Additionally chitin-lignin complexes can be used as adsorbents, due to their high adsorption capacity of numerous toxic metals such as lead, nickel and cadmium^33–35^. Finally, recent studies showed that they could be used for intelligent drug delivery systems as they respond to pH and thermal stimuli^32,36,37^.

Previously, we demonstrated the possibility of generating an ECM-like fibrous scaffold from chitin-lignin gels by electrospinning. Additionally, we reported significantly improved mechanical properties and antimicrobial performance by incorporating a biodegradable, tough elastomer poly(glycerol sebacate) (PGS)^24,32^. However, we found that these scaffolds quickly degrade in water-based media, providing only an immediate burst release of drugs. One of the approaches to overcome such challenges is the encapsulation of these gels by a hydrophobic shell polymer using coaxial electrospinning, which can also be used to generate hollow fibers^16,17,38^. A common hydrophobic polymer, polycaprolactone (PCL) is an aliphatic polyester that has been widely investigated for many biomedical applications, including wound healing, owing to its easy processability and biocompatibility^39,40^. The slower biodegradation rate and moderate hydrophobicity of PCL let it serve as a good barrier to retard fast dissolution of chitin-lignin (CL) gels in water^40,41^.

Employing coaxial electrospinning has become a popular approach to encapsulate and control the drug release in recent years^42,43^. Combining natural gel polymers and synthetic polymers in a core–shell format could further maximize the benefits offered by this technology^43^. For example, Adeli-Sardou et al. encapsulated lawsone loaded gelatin with PCL by coaxial electrospinning in order to decelerate biodegradation and the drug release rate of gelatin. The fabricated scaffold is suggested for wound healing applications due to its structural, mechanical and biochemical characteristics, and excellent biocompatibility^44^. Similarly, Chen et al. applied coaxial electrospinning to wrap chitin derived glucosamine sulfate (GAS) into PCL, in which sustainable release of GAS could promote proliferation and growth of chondrocytes in cartilage tissue regeneration^45^. Nevertheless, entrapping multicomponent gels with several natural and synthetic polymers and drugs by coaxial electrospinning has not yet been attempted.

Here, we developed PCL coated CL-based core–shell fibers by coaxial electrospinning. We evaluated morphological, chemical, thermal and mechanical properties of the core–shell fibrous scaffold. Next, drug release characteristics of the core–shell fibrous scaffold were assessed using methylene blue (MB) as a model drug. Afterwards, penicillin/streptomycin (PS)-loaded substrates were used to test the effectiveness of the drug release against gram-positive and -negative bacterial strains. Finally, biocompatibility of the drug loaded platform was assessed using bone marrow-derived mesenchymal stem cells (BM-MSCs) and NIH 3T3 cells. Taken together, these results indicate that this biomaterial platform has many advantages that could significantly improve existing wound healing and dressing products.

## Results and discussion

**Figure 1:**
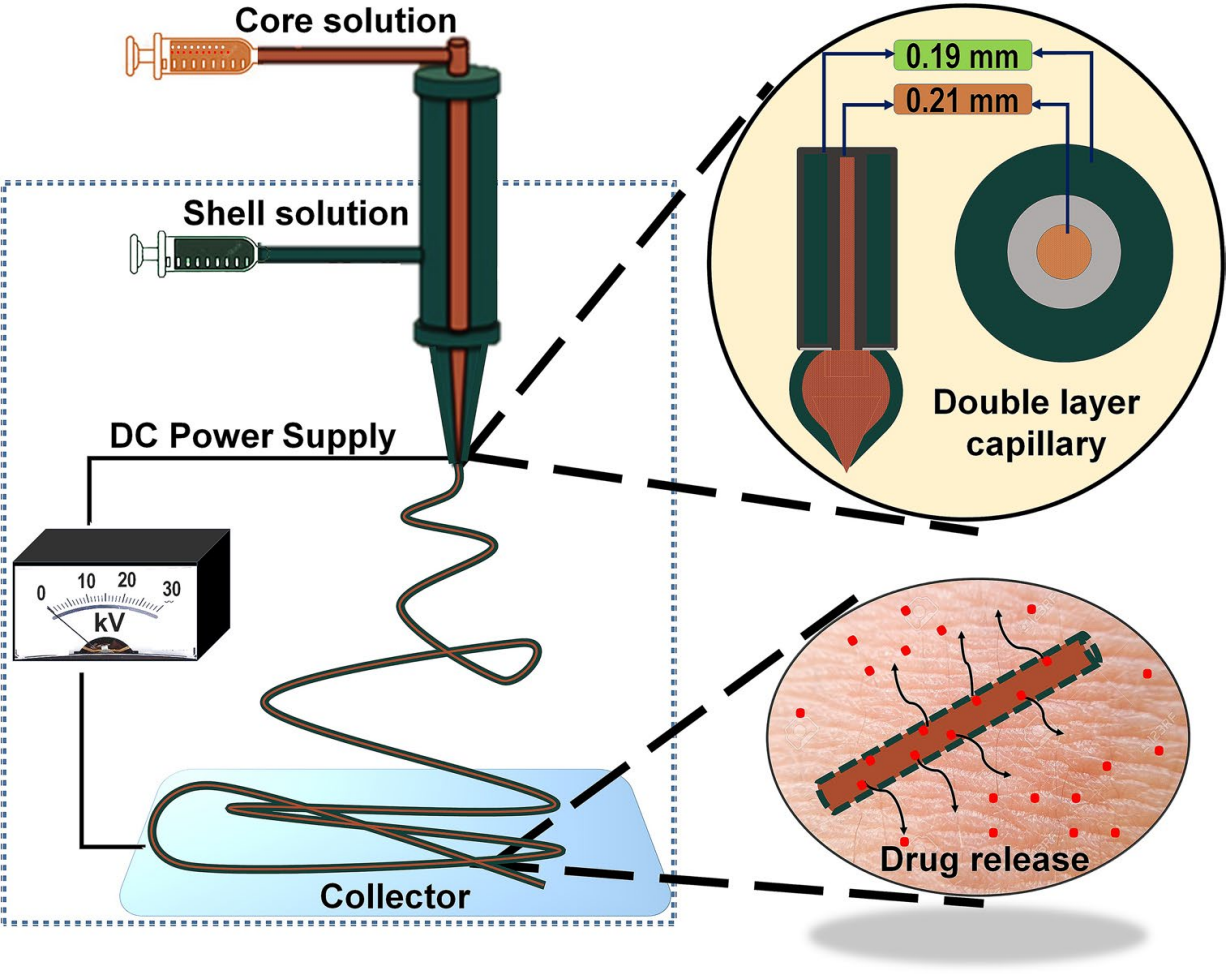
Systematic representation of chitin-lignin (CL) based hybrid fiber encapsulation by PCL using coaxial electrospinning technique and theoretical assumption for its drug release behavior. Here, a mixture of CL sol–gel solution and PGS solution in 9:1 volume ratio is used as a core solution, and PCL solution is used as a shell solution to produce the core–shell fiber. The schematic was prepared by first author Tuerdimaimaiti Abudula.

The fabrication process of core–shell nanofibers using coaxial electrospinning is shown schematically in Fig. 1. The formation of double layered Tailor’s cone takes place from inner and outer droplets fed by different syringes in the presence of a high voltage electrical field^17^. The Tailor’s cone jet latterly stretches towards the collector and as the solvents evaporate it leads to the formation of an ultrathin core–shell fiber^17,46^. Both inner and outer solutions simultaneously experience the same electrical field during this process. Therefore, primary electrospinning parameters including feed rate, polymer concentration, voltage and the nozzle-collector distance need to be optimized and balanced for each solution^47,48^. Previously, we reported 18 kV applied voltage when electrospinning sol–gel CL/PGS (hybrid fiber) solutions^32^. Similarly, 19.5 kV was the applied voltage for electrospinning of a 10 wt.% PCL solution using our in-house system to produce smooth nanofibers^49^. Based on these results, we decreased the PCL concentration to 8 wt.% for the current study in order to achieve stable electrospinning at 18 kV.

In our initial studies, controlling the feed rate during the coaxial electrospinning of core and shell solutions was crucial to achieve smooth core–shell fibers. When the feed rate of PCL solution was too high, the electrospinning process was not stable resulting in significant fusion of fibers. When the feed rate of PCL solution was too low or the feed rate of the CL/PGS solution was too high, the resulting fibers were not effectively encapsulated by PCL. This would ultimately affect in vitro degradation and the controlled drug release. Another factor that had to be considered during electrospinning was the solvent evaporation rate. Specifically, the length of tubes that deliver the polymer solutions needed to be optimized to prevent polymer precipitation and aggregation due to a mismatch between polymer solvent evaporation (i.e. chloroform, solvent for the shell polymer has a much higher vapor pressure than water, solvent for the core solution).

**Figure 2:**
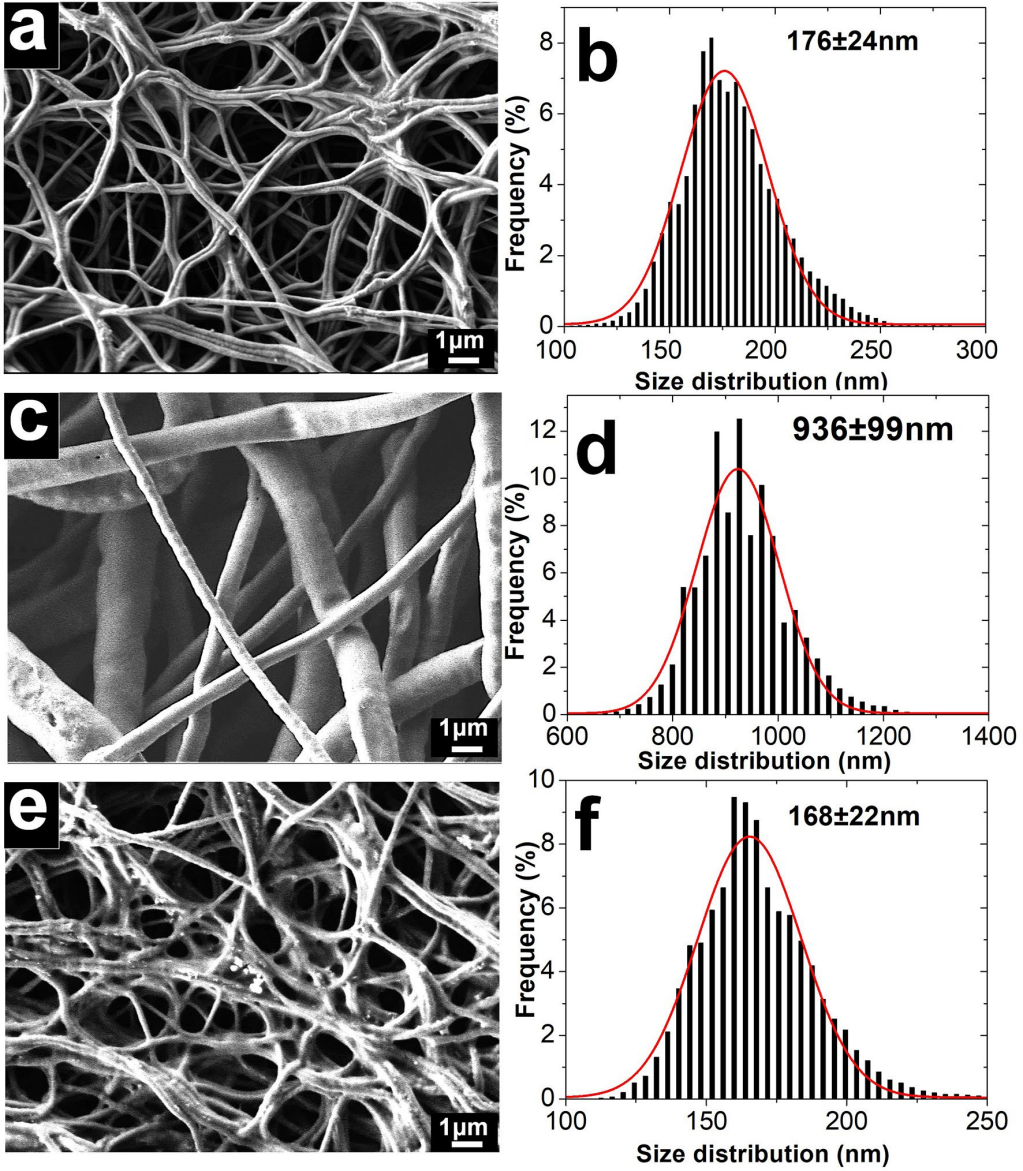
SEM micrographs and fiber size distribution of hybrid fiber **(a,b)**, PCL fiber **(c,d)** and core–shell fiber **(e,f)**. The result shows morphological and dimensional similarity of the core–shell fibers with the hybrid fibers alone.

SEM micrographs and the corresponding fiber size distribution curves of CL/PGS (hybrid), PCL and core–shell fibers are presented in Fig. 2. Although the mean size of PCL fibers is five times higher than hybrid fibers, the produced core–shell fibers showed a similar size distribution as the hybrid fibers alone. This implies that the core layer is the leading contributor to the morphology and size distribution of the core–shell fibers. This might be associated with the high electrical conductivity of the core solution^50^. It was reported previously that the sol–gel CL solution (i.e. 90% of the core solution in our study, the remaining 10% was PGS in our case) has a conductivity of 7.8 miliSiemens (mS)^51^, which is significantly higher than the value for the PCL solution (i.e. almost non-conductive at < 0.04 microSiemens^52^ (µS)). Therefore, high surface charge density might exist that is derived from the core solution in our coaxial setup, which potentially promotes longer elongation of the Tailor cone jet and ultimately increases whipping and decreases fiber size^53^.

**Figure 3:**
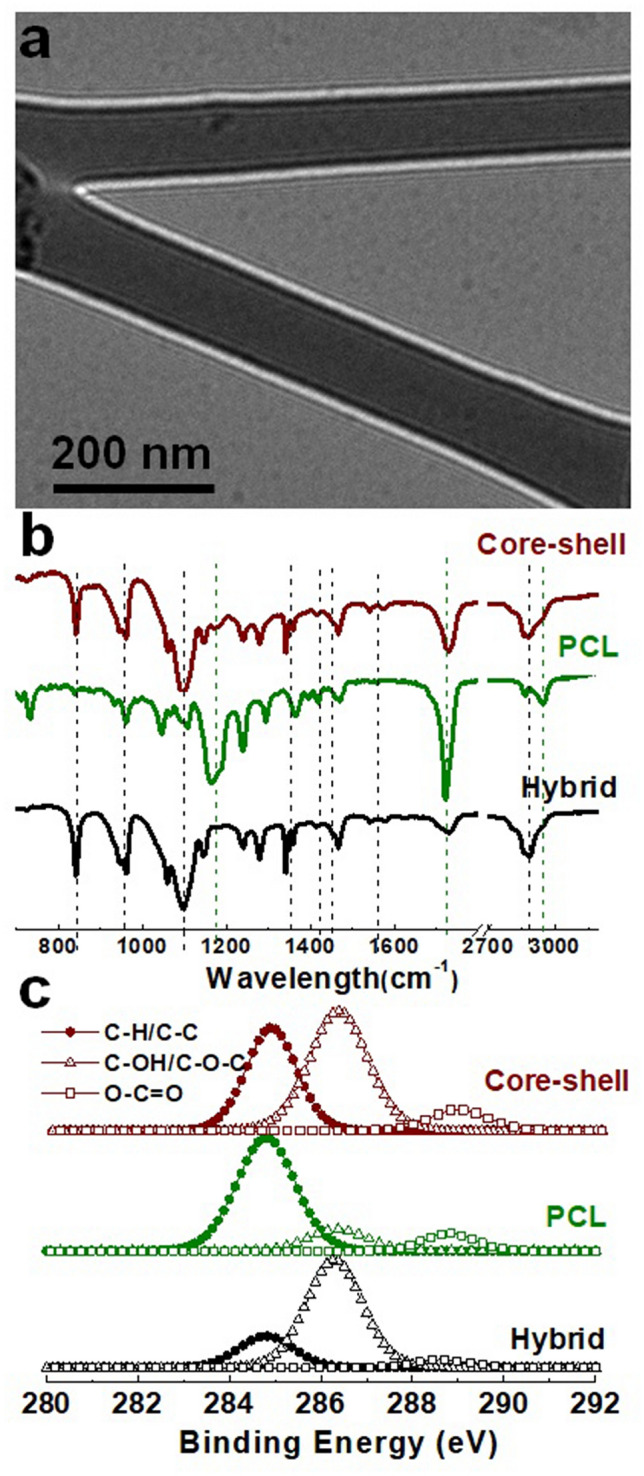
TEM image of the core–shell fiber **(a)**; FTIR spectra of the electrospun scafflds **(b)**, and deconvolved XPS spectra of carbon in the scaffolds **(c)**. Overall results suggests that the shell layer was much thinner compared to the core layer in the resulted core–shell fiber.

TEM imaging was performed to visualize the layered architecture of the core–shell fibers. From the image, it can be observed that clear boundaries existed between core and shell layers, which confirms effective encapsulation of hybrid fibers by PCL (Fig. 3a). We observed that the fiber shell layer was thinner compared to the core layer (Figure S2), which could be attributed to the concentration difference between the core and shell solutions^54^. The core solution not only contained several different polymers with 36.0 wt.% total polymer concentration, but also water partially remained trapped in the core solution to yield a gelatinous fiber core. Namely, density of the resulted core layer (~ 0.371 g/cm^3^ without considering the trapped water) is at least four times higher than the shell layer one (0.0916 g/cm^3^). In addition, slower evaporation rate of the core solvents and its high conductivity increases the inner/core layer thickness when compared to the outer/shell layer during electrospinning^53,55^.

Chemical analysis of the electrospun fibers was performed using FTIR and XPS. The FTIR illustrates bulk composition of the fibrous sheets, while XPS specifically provides their surface layer composition. Detailed analysis of chemical composition of the hybrid and PCL fiber by FTIR have been reported in our earlier studies^32,49^. The FTIR spectra of core–shell fiber appeared to be similar to that of the hybrid fiber, and only strong peaks of PCL such as the peak at ∼ 1723 corresponding to carboxyl stretching (C=O) were noticeable. This emphasizes the dominance of core composition over shell in the core–shell fiber (Fig. 3b). Deconvoluted XPS spectra of carbon for the different fiber samples showed that the spectra corresponded to single bonds between carbon and oxygen (C–O) much more intensely when compared to bonds between carbon and hydrogen (C–H) for the hybrid fibers. This indicates that the C–O bonds are highly dominant in the fiber. On the other hand, peak intensity of the C–H spectra was higher than the C–O spectra in case of PCL fibers, showing dominance of the C–H bond rather than the C–O bond. Comparatively, the XPS spectra of the core–shell fiber indicates a combination of peaks from both the shell and core layers (Fig. 3c). The typical depth of XPS analysis is generally accepted to be up to 10 nm^56^. Thus, the shell thickness of the core–shell fibers being < 10 nm is in agreement with the characterization by both TEM and XPS.

**Figure 4:**
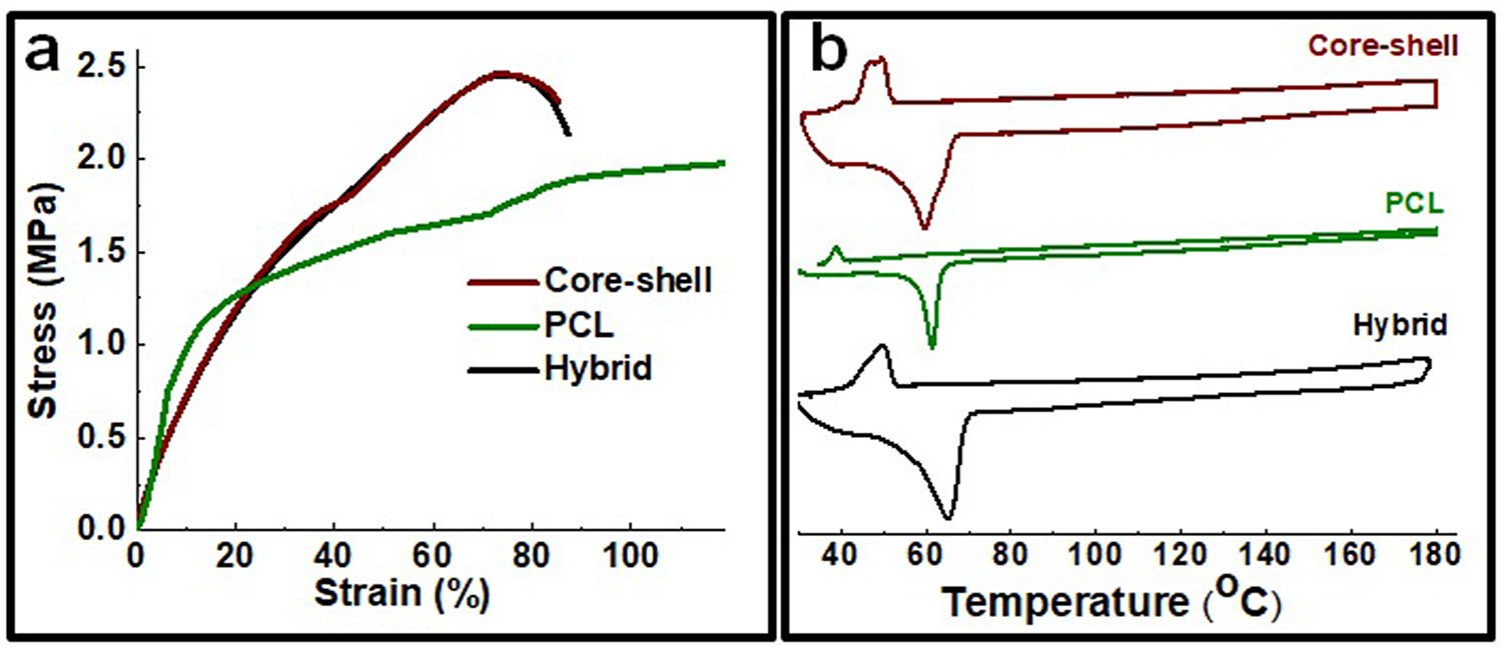
Stress–strain curve **(a)** and DSC curve **(b)** of the electrospun scaffolds. The result indicates insignificant effects of PCL encapsulation on thermal transition and mechanical behavior of the CL based hybrid fibrous scaffold.

Considering that PCL formed a very thin outer layer, it did not considerably influence mechanical behavior of core fibers (Fig. 4). Generally, PCL fibers exhibited similar tensile strength but much higher flexibility compared to the hybrid fibers (Figure S3). No significant change in mechanical compliance was observed for the core–shell fibers. Differential scanning calorimetry (DSC) measurement shows that the endothermic peak of core–shell fibers was a combination of hybrid and PCL fiber peaks, and represents melting points of polyethylene oxide (PEO) (present in the core solution) and PCL respectively (Fig. 4b)^32,57^. Two separate exothermic peaks were observed for core–shell fibers in the cooling process. This might be due to the melting of the core–shell fiber and phase separation during heating, with two distinct crystallizations observed for PEO and PCL in the fibrous mesh.

**Figure 5:**
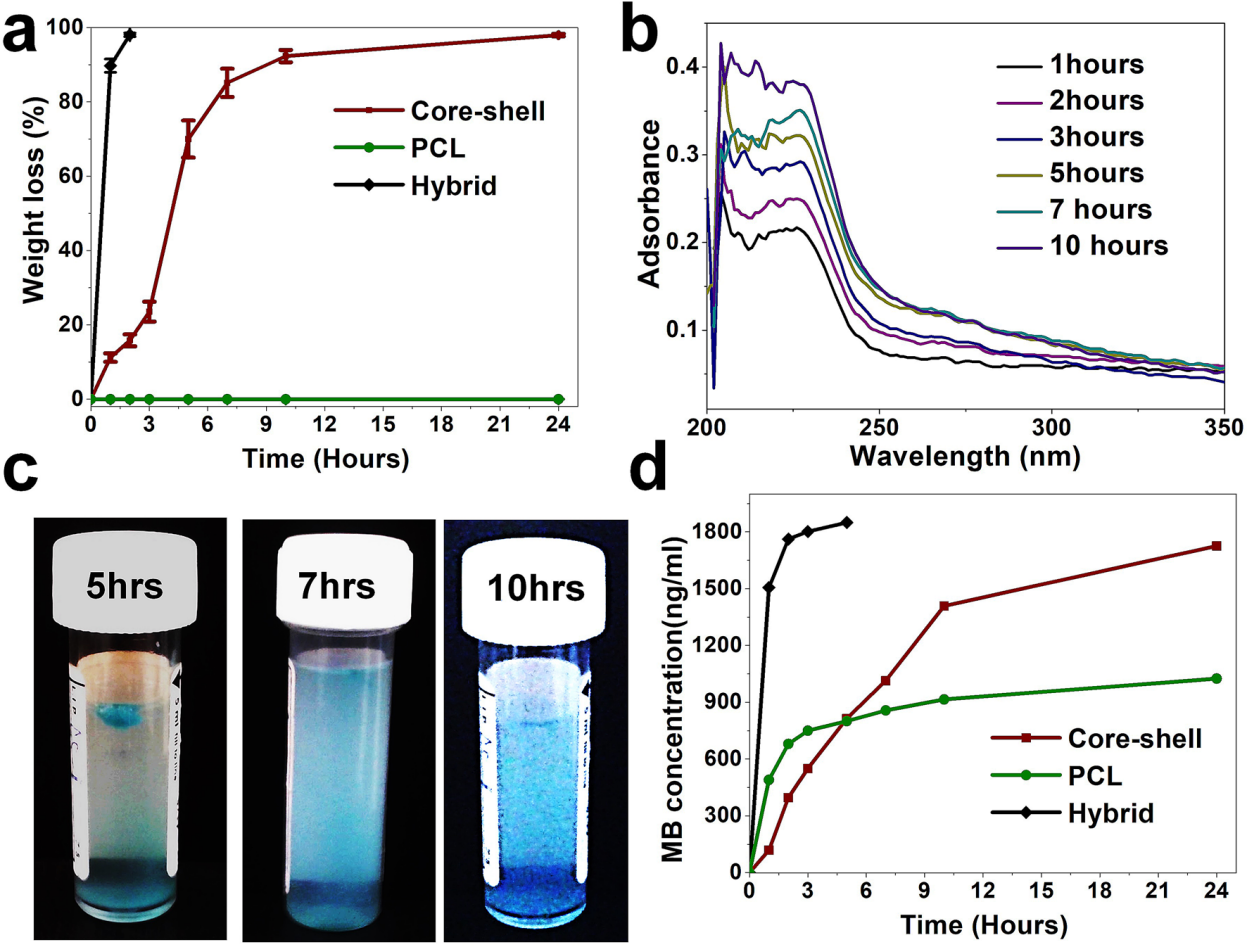
Weight change of the electrospun fiber under PBS (pH 7.4) **(a)**, UV spectra change and representative color change of the PBS solution after immersing the core–shell fibrous sheet **(b,c)**, and methylene blue release profile from the electrospun scaffolds **(d)**. The photo was taken by first author Tuerdimaimaiti Abudula. Overall results imply the role of PCL shell layer coating on the CL based hybrid scaffold in preventing burst release and providing prolonged drug release profile.

The presence of the PCL shell significantly retarded the dissolution rate of the hybrid fiber in PBS media. We found that the CL fibers without PGS dissolved very quickly (i.e. in less than 15 min), and PGS incorporation extended the fiber shelf life for up to 2 h (Fig. 5). However, the core–shell fibers appeared to have a three-stage dissolution profile and shelf life lasted for more than 24 h. The dissolution rate of the core–shell fiber in the initial 3 h was slow, then rapid dissolution occurred in the next 2 h, and it slowed down again in the last stage (Fig. 5a). This is a common phenomenon for many biodegradable polymers, which could be associated with diffusion effect of PBS through hydrophobic PCL layer, interfacial diffusion between PBS and the core layer of the fiber and molecular weight change of the individual polymers^58,59^. UV spectra of PBS media after immersing the core–shell fiber exhibited a broad absorbance in the range of 200–230 nm, and its intensity directly increased with immersion time (Fig. 5b). Liu et al.^60^ suggested that *N*-acetylglucosamine and glucosamine residues in chitin were UV chromophoric, and they showed a broad, high extinction coefficient under 225 nm wavelength. According to Shende et al.^61^ unsaturated chains of lignin are capable of adsorbing UV light at about 210 nm wavelength. Accordingly, these results suggest that dissolution of chitin and lignin mostly follows the overall dissolution of core layer in core–shell fiber.

Methylene blue (MB) as a model drug was used to examine the drug release behavior of the core–shell fibers. MB is a multifunctional therapeutic agent, and it has a century of medical practice history^61–64^. MB could strongly inhibit nitric oxide, which abates endothelial function, restrains synaptic transmission, moderates immunity, and causes cell death by activating guanylate cyclase^65,66^. MB is also a strong antioxidant, which can protect cells and tissues from the noxious effects of reactive oxygen species by competing with molecular oxygen for the electron transferred by xanthine oxidase^65,67^. Additionally, activation, adhesion and aggregation of blood platelets can be prevented by MB^65^. Moreover, MB has also been used as a photosensitizer in photodynamic therapy to positively modulate the vascular wound healing response^68^.

MB is also a photodynamic dye, and its concentration dependent color change at very low concentration ranges can be visualized. Therefore, we used image processing to determine the concentration change of MB after immersing the scaffolds into PBS media. A mathematical model was devised (see “Methods” section) according to the relative intensity change between red, green and blue (RGB) colors following calibration with a known concentration of MB (Figure S1). Visual images of the PBS after immersing the core–shell fiber is given in Fig. 5c, and relative concentration of MB, by image processing is shown in Fig. 5d. The results indicate that both hybrid and PCL fibers experience different levels of initial burst release. However, in case of core–shell fibers, the release of MB was directly proportional to the dissolution rate of the core fiber layer. This suggests the PCL shell layer coating prevents burst release and provides a prolonged drug release profile.

**Figure 6:**
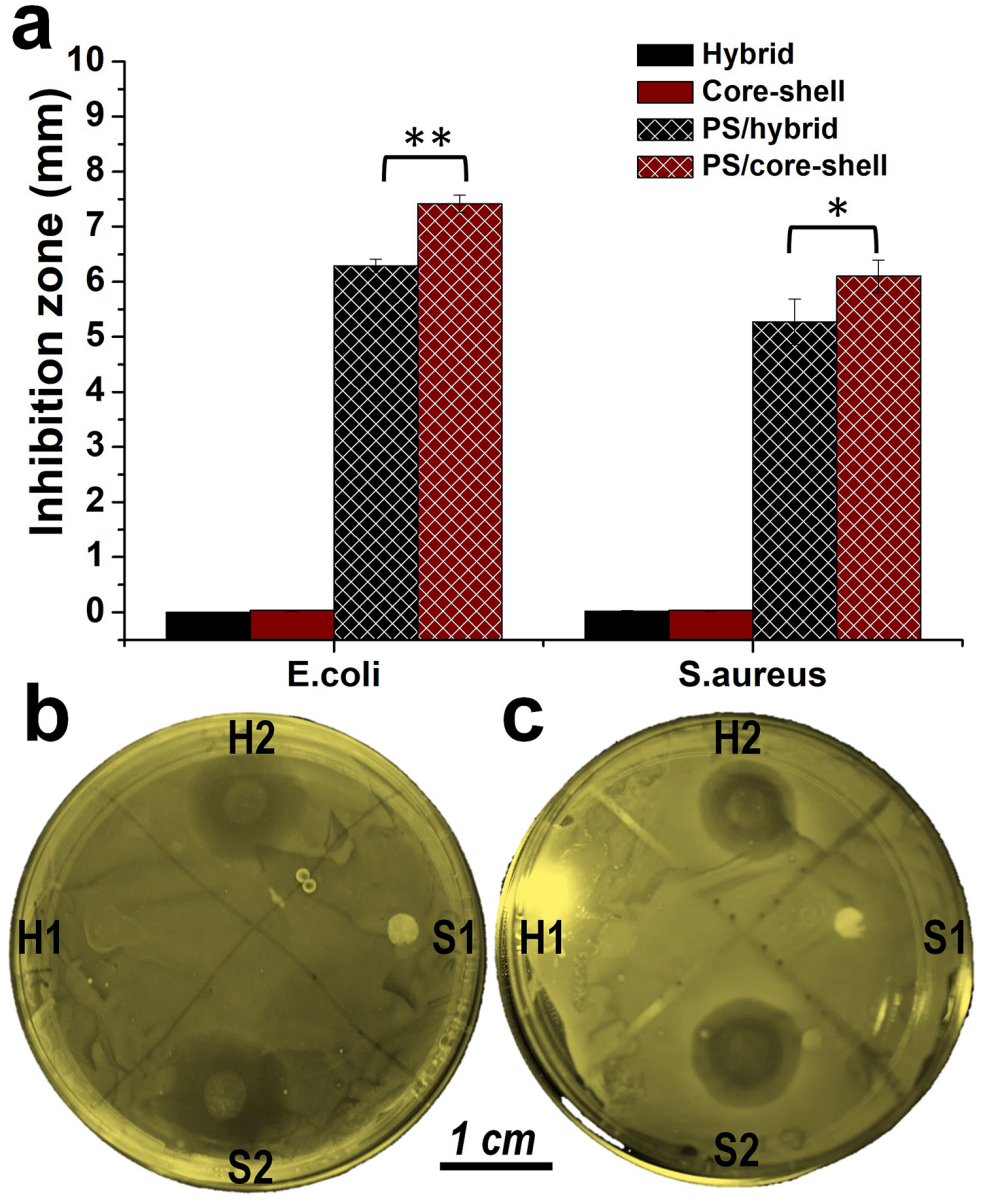
Inhibition zone of the PS loaded fibrous scaffolds against E.coly and S.arous. **(a)** Diameter of the inhibition zone, calculated according to triple experiment. **(b)** Representative inhibition zone of the scaffolds against E.coly. **(c)** Representative inhibition zone of the scaffolds against S.arous. Where H1 and S1 represents hybrid and core–shell fibrous scaffold, H2 and S2 represents PS loaded hybrid and core–shell fibrous scaffold. PS loaded core–shell fibrous scaffolds showed better antibacterial performance than that of hybrid scaffolds due to controlled release of the antibiotics. The photo was taken by first author Tuerdimaimaiti Abudula.

The effect of drug release behavior was tested using bacterial inhibitory effect of penicillin/streptomycin (PS) loaded into the substrates against the common pathogens *E. coli* and *S. aureus*. Both PS loaded hybrid scaffold and core–shell fibrous scaffolds showed a clear inhibition zone against *S. aureus* and *E. coli* bacterial strains. Core–shell fibrous scaffolds showed a superior inhibition effect, compared to that of hybrid scaffold against both pathogens (Fig. 6). This implies controlled release of antibiotics could improve their inhibitory potential against bacterial strains by maintaining a constant and localized release of therapeutics^2,3^. Both scaffolds showed a smaller inhibition zone against *S. aureus* compared to *E. Coli*. More interestingly, a double zone of inhibition was observed in case of *S. aureus*, in which the inner zone was darker than the outer one (Fig. 6b,c), indicating that tolerance of *S. aureus* to PS was higher than for *E. Coli*^69,70^.

**Figure 7:**
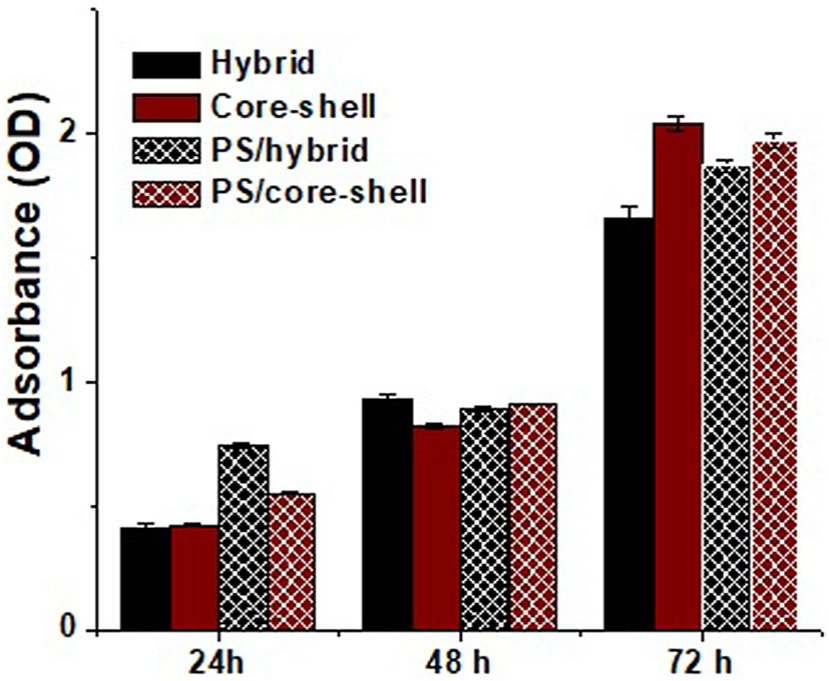
MTT assay following culture of bone marrow mesenchymal stem cells (BM-MSCs) for 24, 48 and 72 h. Individual fibers show increases in cell proliferation with time. The values are expressed as mean ± SD from three independent experiments. All the scaffolds showed a good biocompatibility, and the incorporation of PS as an antibiotic within PCL coated hybrid scaffolds did not show any significant negative effects on the cell proliferation.

In most in vitro wound healing studies, dermal fibroblast cells are used. However, recent literature suggests that bone marrow-derived mesenchymal stem cells (BM-MSCs) have a number of advantages including higher re-epithelialization rate, better cell infiltration, and angiogenic effects^71,72^. However, BM-MSCs typically have a lower tolerance to reagents in comparison to fibroblasts. Therefore, we tested cytocompatibility of PS loaded scaffolds using BM-MSCs in vitro. Furthermore, in clinical applications patient’s own cells could be utilized for developing cell laden scaffolds and dressings leading to improved chronic non-healing wound treatment. As shown in Fig. 7, the adhesion and growth of BM-MSCs were observed with both PS loaded and control scaffolds through 72 h. There were no significant differences in cell proliferation between the control and loaded scaffolds. The incorporation of PS within PCL coated hybrid scaffolds did not affect cell proliferation on the produced fiberous mats^73^. Similarly, using NIH 3T3 cells we observed minimal negative viability effect. Specifically, the proliferation rate of cultured 3T3 cells in the presence of various electrospun scaffolds was assessed by PrestoBlue Cell Viability Reagent at 24 h of culture (Figure S4). The results showed similar metabolic activity and proliferation between scaffolds and control cells during the culture period. As such, these core–shell fibrous scaffolds could serve as novel biomaterial based wound dressings with sustained controlled drug release that could improve healing and treatment outcomes.

## Conclusion

Scaffolds formed from core–shell fibers were fabricated using co-axial electrospinning in which the core was a hybrid gel of CL/PGS surrounded by a PCL shell. SEM results showed that the hybrid fibers preserved their morphological integrity after coating. It was confirmed by TEM and XPS that the PCL shell layer was much thinner than the core layer. Therefore, the presence of PCL encapsulation did not significantly influence thermal or mechanical properties of the core fiber. However, a significant retarding effect of PCL coating on dissolution rate of the hybrid fiber was observed. Ultimately, the PCL coated hybrid fibers had a much longer shelf life and provided sustainable drug release. Antibiotics were successfully loaded into the core–shell fibers and their release showed superior antibacterial effects against common bacterial pathogens found on skin without causing any observable cytotoxicity. Taken together, these core–shell based biomaterials could lead to the design of novel drug releasing biomaterials for wound dressing and healing applications. More importantly, these results might stimulate wider interest and application of natural polymers derived from food and agriculture by-products.

## Methods

### Materials and solution preparation

Chitin nanofibrils in the form of 2% water suspension, bio-lignin (CIMV, France) and PEOX were provided by Nanoscience Centre (MAVI, Italy). All the other chemicals were purchased from Sigma-Aldrich (St. Louis, MO, USA). Polyglycerol sebacate (PGS) was prepared using polycondensation of glycerol and sebacic acid in 1:1 ratio as reported previously^74^.

The hybrid solution was prepared by mixing CL sol–gel solution and PGS solution in 9:1 volume ratio, with gentle stirring for 30 min. Details of CL based sol–gel solution and PGS solution preparation have been reported in our earlier studies^32^. Briefly, 30 wt.% of chitin nanofibrils suspension, 0.1 wt.% of bio-lignin, and 7 wt.% of PEOX were dispersed into 62.9 wt.% deionized water, pH of the mixture was raised to 10.5 using 0.1 M of NaOH. Then, the mixture was shielded and kept on magnetic stirrer for 48 h to obtain a uniform CL sol–gel solution. PGS solution was prepared by dissolving 25% (w/v) of PGS in ethanol under stirring for a half hour.

8% (w/v) of PCL was dissolved in chloroform and ethanol (9:1) by stirring at room temperature for 3 h.

### Coaxial electrospinning

NANON-01A Electrospinning System (MECC, Fukuoka, Japan) was adapted for coaxial electrospinning under normal lab atmosphere with 63% humidity. The PCL solution was delivered to the outer layer of Ultra-thin coaxial spinneret (NANON Supply, MECC, Fukuoka, Japan) by system provided syringe pump using Teflon tube (Cole-Parmer Instrument Company, Vernon Hills, IL, USA) at 0.5 mL/h of feed rate. An extensional syringe pump (KDS 100, KD Scientific Inc, USA) connected with 20 cm of Teflon tube was used to deliver the hybrid solution into a 27-gauge blunt metallic needle (NANON Supply, MECC, Fukuoka, Japan) at 0.2 mL/h of feed rate. Then the double layer solution was electrically stretched at18 kV of voltage over 150 mm of distance. For individual hybrid fiber and PCL fiber preparation, feed rate was fixed at 0.3 mL/h and 0.9 mL/h respectively. During the electrospinning, the needle was allowed to axially sway in 8 cm range with 10 cm/s of moving speed, and the formed fibers were collected on a stationary flat aluminum sheet. Electrospinning was performed for 4 h in all cases, and the needle tip was automatically cleaned every 5 min. The collected samples were dried for 48 h at room temperature before any characterization test.

### Characterization

Microstructure of the electrospun membranes was observed using JSM 7600F scanning electron microscopy (FESEM, JEOL, Japan). The fiber size distribution was determined using a newly developed digital image processing method in “Matlab”, which generates statistically reliable Gaussian distribution curves with punctilious density estimation. Details of the image processing method can be found elsewhere^75^.

Layer structure of core–shell fiber was observed by JEM-2100 F high-resolution transmission electron microscope (FETEM, JEOL, Japan). The fiber was electrospun for 45 s on a carbon holey grid for TEM sample preparation. Shell thickness of the core–shell fiber was estimated using direct measuring method on “Image J” software, based on 12,000-magnified TEM image. 100 images data were obtained from different positions of the fiber to determine mean shell thickness of the fiber.

Bulk composition of the fibrous mesh was dissected based on Attenuated Total Reflected Fourier transform infrared spectroscopy (ATR-FTIR, Thermo Fisher Scientific, MA, USA) in 400–4000 cm^−1^ of wavenumber range. Surface composition of the prepared fibers was analyzed by X-ray Photoelectron Spectroscopy (XPS) measurements using a surface nano analysis system (SPECS GmbH, Germany). SPECS XR-50 with Mg-Kα at1283.6 eV was used to irradiate the sample. 284.6 eV corresponding to C–C bond was used as a reference to determine binding energies of elements contained in the sample.

Differential scanning calorimetry (DSC-60, MICRO DSC3 EVO, Setaram Inc, USA) was used to examine thermal behavior of the samples. Endothermal and exothermal curves were obtained by heating the fibrous sheets up to 180 °C, then cooling them down to 25 °C. Nitrogen 35 mL/min of feed rate was used as purgent. Uniaxial tensile test measurement for the fibrous mat (4 cm in length and 1 cm in width) was conducted using universal tensile machine (Lloyd Instruments Ltd., Bognor Regis, UK). The tensile curve was obtained by stretching the sample at 10 mm/min extension speed, and the sample thickness was measured by electronic caliper.

### Biodegradation and drug release

An in vitro biodegradation test and methylene blue (MB) release test was performed by immersing 20 mg of the electrospun fibrous mats into 5 mL of phosphate buffer saline (PBS) at 37.5 °C. MB was incorporated into the core solution at 2 mg/mL of concentration before electrospinning. The weight of the mats over different periods of time was measured by micro balance (0.01 mg precision) and a UV test was performed for the solution using UV–Vis spectrophotometer (NanoDrop 2000, Thermofisher Scientific, USA). Color change of the solution was also recorded by using digital camera. Image processing technique in Matlab software (R2017a) was used to analyze the MB release behavior in fibrous mat. Carefully cropped solution image matrix was divided into red, green and blue (R,G,B) channels, and average intensity of each channel was calculated. Finally, the concentration of MB in the solution was correlated with average intensity of RGB channel by the following equation (See Table S1 and Figure S1):

1 $${C}_{MB}=41.202 {(G-R)}^{0.95}+319.05 , \left({R}^{2}=0.9766\right)$$

where ${C}_{MB}$ is concentration of MB in the solution (ng/mL), G and R are the average intensity of the green and red channels.

### Antibacterial activity and biocompatibility

Loading of PS was achieved by introducing 1000 unit/mL of penicillin and 1000 µg/mL of streptomycin in the core solution and electrospinning at similar conditions as mentioned above. Antibacterial activity of the samples was evaluated by agar diffusion test, as mentioned in our previous literature^57^. Isolated colonies of E. coli strain and S. aureus strain were placed in Lysogeny broth (LB) media containing ampicillin (100 μg/mL) at 37 °C, and cultured overnight. Then 200 μL of cultured strains were spread on the LB-agar plate surface containing 100 μg/mL of ampicillin to get a mat of bacteria. Disks of hybrid and core–shell fibrous scaffold (three for each) containing PS were placed on the top of bacteria strain and incubated overnight. Non drug loaded scaffolds were used as control.

Biocompatibility of PS loaded scaffolds were tested by culturing bone marrow-derived mesenchymal stem cells (BM-MSCs) as described in our previous work with some modification^76^. Sterilized PS loaded scaffolds (~ 2 mg dry weight) were cut and put in a 48-well culture plate. These scaffolds were then seeded with BM-MSCs (1 × 10^4^ cells/well), and the culture plate incubated under regular cell culture conditions in a 5% CO2 incubator at 37 °C. 3-(4,5-dimethylthiazolyl-2)-2,5-diphenyltetrazolium bromide reagent kit (MTT) was used as cell proliferation assay. 0.5 mg/mL MTT reagent was introduced to the cells in culture and the culture plate was incubated in a 5% CO2 incubator until the purple formazan precipitate was formed. Then 100 μl of the detergent reagent was used to dissolve the formazan in the dark for 2 h. Optical density of the formazan was measured by a microplate reader (SpectraMax i3, San Jose, USA) at 570 nm of wavelength using 630 nm of wavelength as a reference.

To quantify the viability and proliferation rate of NIH 3T3 cells measurement of their metabolic activities using PrestoBlue Cell Viability Reagent (Invitrogen) was performed 24 h after culturing while the samples were kept and maintained in complete growth media as was described in our previous study.^77^ Specifically, in the incubator at 37 °C and for 1 h we placed and incubated the samples in a solution of 10% (v/v) of Prestoblue reagent in growth media. The fluorescence intensity of the solution was measured using a Cytation 5 Cell Imaging Multi-Mode Reader (Biotek, USA) at 540 nm (excitation)/600 nm (emission).

### Statistical analysis

Statistical analysis was performed in Origin (OriginPro 8.0, Origin Lab Inc., USA) using One-way Analysis of variance (ANOVA) approach. Statistical significances at p < 0.05 and p < 0.01 was nominated by single and double asterisks respectively.

## Supplementary information

Supplementary Information.
