# Author Correction: Sustainable drug release from polycaprolactone coated chitin-lignin gel fibrous scaffolds

**DOI:** 10.1038/s41598-022-19505-w

**Published:** 2022-09-05

**Authors:** Turdimuhammad Abdullah, Kalamegam Gauthaman, Azadeh Mostafavi, Ahmed Alshahrie, Numan Salah, Pierfrancesco Morganti, Angelo Chianese, Ali Tamayol, Adnan Memic

**Affiliations:** 1 Center of Nanotechnology, King Abdulaziz University, Jeddah, Saudi Arabia; 2 Center of Excellence in Genomic Medicine Research, King Abdulaziz University, Jeddah, Saudi Arabia; 3 Faculty of Medicine, AIMST University, Semeling, Bedong, Kedah Malaysia; 4 Department of Mechanical and Materials Engineering, University of Nebraska, Lincoln, NE USA; 5 Academy of History of Healthcare Art, Rome, Italy; 6 ISCD Nanoscience Center, Rome, Italy; 7 LABOR Srl, Rome, Italy; 8 Department of Biomedical Engineering, University of Connecticut Health Center, Farmington, CT 06030 USA

Correction to: *Scientific Reports* 10.1038/s41598-020-76971-w, published online 24 November 2020

Since the publication of this work, Turdimuhammad Abdullah has changed their name from Tuerdimaimaiti Abudula.

The original HTML and PDF versions of this Article, and its accompanying Supplementary Information file have been corrected.

