# Supplementary material for: Sustainable drug release from polycaprolactone coated chitin-lignin gel fibrous scaffolds
Source: Sci Rep. 2020 Nov 24;10:20428. doi: 10.1038/s41598-020-76971-w (PMC7686307; doi:10.1038/s41598-020-76971-w)
Supplement: Supplementary file 1 — Supplementary Information. [file 41598_2020_76971_MOESM1_ESM.docx]

Sustainable drug release from polycaprolactone coated chitin-lignin gel fibrous scaffolds

**Turdimuhammad Abdullah^1^, Kalamegam Gauthaman^2,3^, Azadeh Mostafavi^4^, Ahmed Alshahrie^1^, Numan Salah^1^, Pierfrancesco Morganti^5^, Angelo Chianese^6^, Ali Tamayol^4,7^ and Adnan Memic^1^***

^1^Center of Nanotechnology, King Abdulaziz University, Jeddah, Saudi Arabia

^2^Center of Excellence in Genomic Medicine Research, King Abdulaziz University, Jeddah, Saudi Arabia;

^3^Faculty of Medicine, AIMST University, Semeling, Bedong, Kedah, Malaysia

^4^Department of Mechanical and Materials Engineering, University of Nebraska, Lincoln, NE, USA

^5^Dermatology Department, 2^nd^ University of Naples, Italy; R&D Director, Nanoscience Centre MAVI, Aprilia (LT), Italy

^6^Chemical Materials Environmental Engineering Department, Sapienza University of Rome, Rome, Italy

^7^Department of Biomedical Engineering, University of Connecticut Health Center, Farmington, CT, 06030 USA

***Correspondence:** amemic@kau.edu.sa

Table S1: Average intensity change between red, green and blue channel at different concentration of methylene blue

| Methylene blue Concentration | Average intensity of channels | | | (G-R)^0.95^ |
| --- | --- | --- | --- | --- |
|  | Red(R) | Green (G) | Blue (B) |  |
| 400 ng/ml | 232.98 | 233.37 | 234.17 | 0.412116 |
| 800 ng/ml | 165.28 | 182.60 | 188.92 | 15.01519 |
| 1200 ng/ml | 170.42 | 199.26 | 209.18 | 24.3768 |
| 1600 ng/ml | 198.57 | 234.1 | 230.91 | 29.71031 |
| 2000 ng/ml | 141.55 | 184.81 | 180.38 | 35.83399 |
| 2800 ng/ml | 71.59 | 148.34 | 151.49 | 61.77426 |

Figure S1: Numerical correlation between MB concentration and Green-Red channel intensity difference in the solution


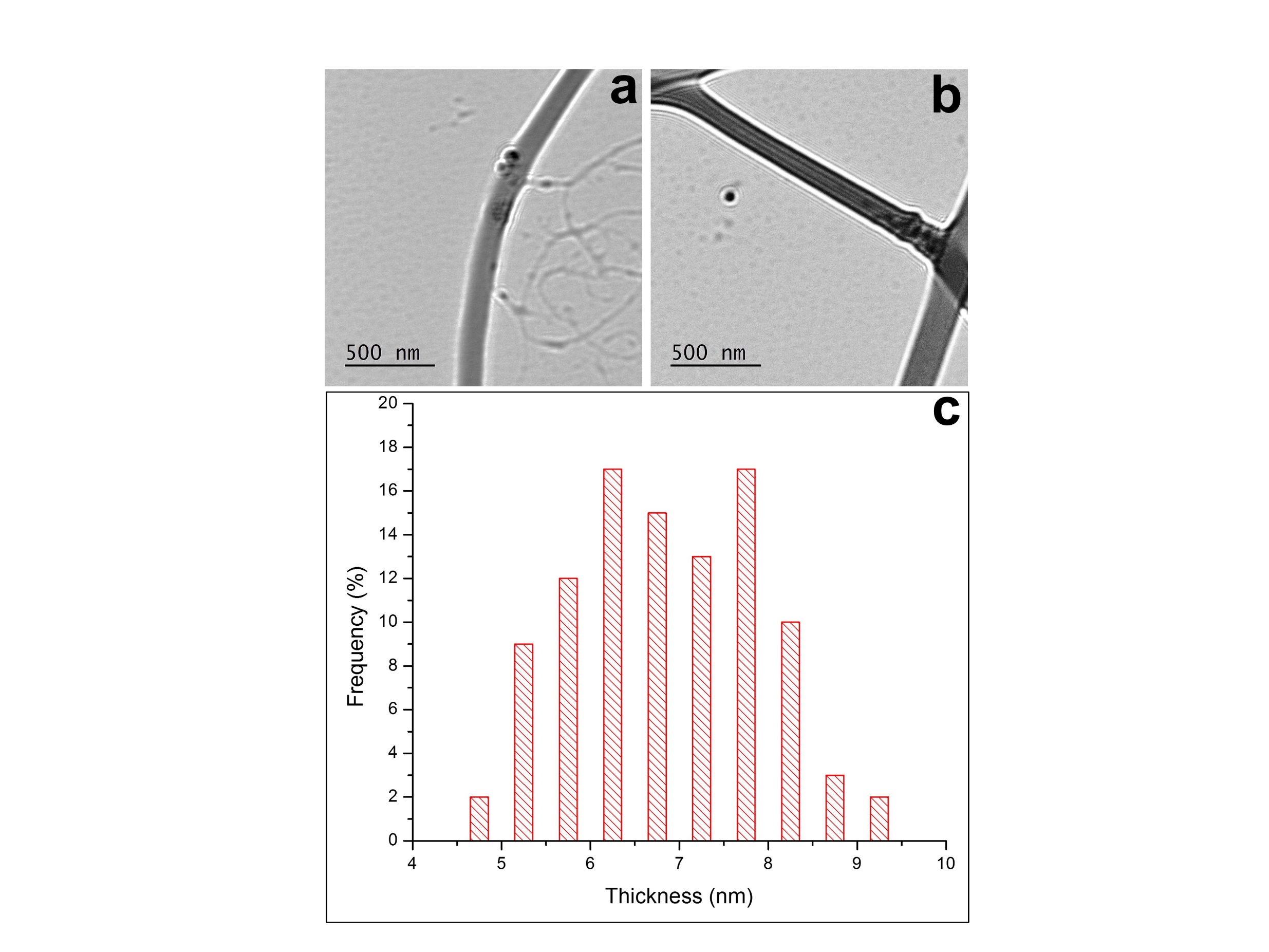


Figure S2: TEM image of the hybrid fiber (a) and the core-shell fiber (b), and the calculated shell thickness distribution (c)

**Figure S3:** Stress-strain curve for electrospun PCL scaffold

**Figure S4:** Viability of NIH 3T3 fibroblasts expressed by NIH 3T3 fibroblasts after culturing with the scaffolds for 24 hours.
